# Deviations from temporal scaling support a stage-specific regulation for *C. elegans* postembryonic development

**DOI:** 10.1186/s12915-022-01295-2

**Published:** 2022-04-27

**Authors:** Alejandro Mata-Cabana, Francisco Javier Romero-Expósito, Mirjam Geibel, Francine Amaral Piubeli, Martha Merrow, María Olmedo

**Affiliations:** 1 Departamento de Genética, Facultad de Biología, Universidad de Sevilla, Avenida Reina Mercedes s/n, 41012 Seville, Spain; 2 Faculty of Medicine, Institute of Medical Psychology, LMU Munich, Goethestrasse 31, 80336 Munich, Germany; 3 Departamento de Microbiología y Parasitología, Facultad de Farmacia, Universidad de Sevilla, Profesor García González, 41012 Seville, Spain

**Keywords:** Development, Timers, Developmental rate, Scaling, Temperature, Arrhenius, Nutrients, Insulin signalling

## Abstract

**Background:**

After embryonic development, *Caenorhabditis elegans* progress through for larval stages, each of them finishing with molting. The repetitive nature of *C. elegans* postembryonic development is considered an oscillatory process, a concept that has gained traction from regulation by a circadian clock gene homologue. Nevertheless, each larval stage has a defined duration and entails specific events. Since the overall duration of development is controlled by numerous factors, we have asked whether different rate-limiting interventions impact all stages equally.

**Results:**

We have measured the duration of each stage of development for over 2500 larvae, under varied environmental conditions known to alter overall developmental rate. We applied changes in temperature and in the quantity and quality of nutrition and analysed the effect of genetically reduced insulin signalling. Our results show that the distinct developmental stages respond differently to these perturbations. The changes in the duration of specific larval stages seem to depend on stage-specific events. Furthermore, our high-resolution measurement of the effect of temperature on the stage-specific duration of development has unveiled novel features of temperature dependence in *C. elegans* postembryonic development.

**Conclusions:**

Altogether, our results show that multiple factors fine tune developmental timing, impacting larval stages independently. Further understanding of the regulation of this process will allow modelling the mechanisms that control developmental timing.

**Supplementary Information:**

The online version contains supplementary material available at 10.1186/s12915-022-01295-2.

## Background

*Caenorhabditis elegans* postembryonic development consists of four larval stages (L1 to L4). At the beginning of each larval stage, a nutritional checkpoint controls initiation [1]. Towards the end of each larval stage, molting occurs. The initiation of each molt is marked by the sealing of the buccal cavity of the larva, which impedes food intake [2]. During the molts, larvae enter a quiescent sleep-like state [3]. The end of each molt is determined by the removal of the old cuticle, called ecdysis, thus concluding that larval stage. Molts and intermolts are therefore fundamentally different processes within each larval stage. Unfortunately, most measurements of postembryonic development do not allow precise resolution of the timing of molts and intermolts.

The repetitive process of molting is coupled to oscillatory expression of ~3700 genes [4–6]. One of the oscillating genes is *lin-42*, the homologue of the *Drosophila* melanogaster clock protein period [7]. LIN-42 has a reiterative function during larval development that is reminiscent of the cyclic functions of clock proteins [8]. On this basis, *C. elegans* larval development has been compared to other rhythmic processes, especially daily or circadian rhythms [6, 8, 9], (reviewed in [10]). However, the four larval stages have non-identical durations [6, 8, 11–13] and are characterized by stage-specific patterns of cell division [14]. The stage-specific fates are controlled by the heterochronic gene pathway. Heterochronic mutants show alterations in the normal order of stage-specific cell division patterns throughout the animal [15]. These mutants showed that the stage-specific patterns of cell division are modular, that is, that events in one larval stage can be independent from events at the previous stage [16]. At the level of the oscillations in gene expression, each stage is also modular. Once transcription of oscillating genes is activated at each larval stage, it progresses until the end of the larval stage [6, 17].

Although the progression of development is under strict genetic control [16], the speed of the process depends on a variety of environmental factors. A central question remains as to how the overall speed of postembryonic development is modulated by rate-limiting interventions such as reduced temperature or poor nutrition. Rate-limiting interventions could act by reducing the overall speed of a timer (or timers) that controls the development completely. Then, these interventions would have similar effects in all stages of development, yielding slower or faster animals, with larval stages that scale proportionally to the total duration of development. In contrast, rate-limiting interventions could stop the timer or reduce its speed only at certain stages of the developmental process. In this scenario, different interventions might independently impact the events taking place during each stage of development. This way, perturbations that affect overall duration of development may have different impact on different stages, depending on the specific events that take place during each stage. The solution to this question calls for precise quantification of postembryonic development in response to varied environmental perturbations.

The speed of both embryonic and postembryonic development in *C. elegans* is temperature sensitive, as in many poikilothermic organisms. For instance, postembryonic development can be completed in about 39 h at 25 °C compared with 75 h at 16 °C [12]. Changes in the bacterial diet and in insulin signalling also affect developmental rate [18–21]. Both of these interventions may increase or decrease metabolic rates and available energy thus liberating more or less energy that can be applied to development. Although temperature, diet and insulin all impact metabolism, they presumably do so via distinct pathways: altered biochemical rates of reactions due to temperature, altered nutrient availability or energy metabolism as regulated by food quantity and quality.

In this work, we have imposed environmental and genetic perturbations to characterize the stage-specific response of *C. elegans* postembryonic development using a quantitative high-throughput method. We quantified development of ~2500 individually assayed larvae. Importantly, we monitored development continuously, with a time resolution of 5 min, and resolved the transitions between molts and intermolts. We analysed interindividual variability of development, its dependence on temperature and food, and its regulation by insulin signalling. In all cases, we observe that interventions that alter developmental timing have a differential effect on the discrete stages of larval development. Furthermore, modification of the cell division pattern by downregulation of heterochronic genes suggests that the duration of the stages might be modulated by the divisions taking place during each stage. These observations support independent regulation of the duration of each larval stage, resulting in the lack of time scaling of the postembryonic *C. elegans* development.

## Results

### Quantitative analysis of *C. elegans* postembryonic development

**Fig. 1:**
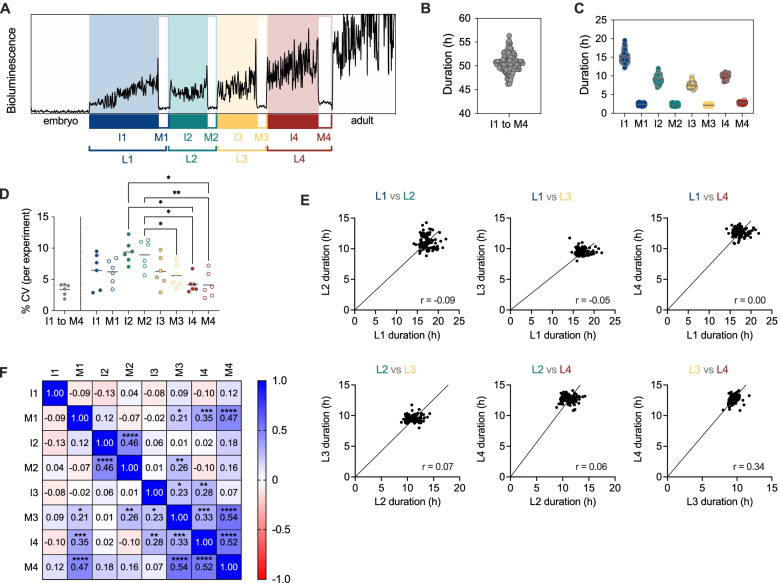
Quantitative analysis of development in 103 individual larvae. **A** Nomenclature of the different stages of development as defined by the luminometry assay. **B** Total duration of development, from hatching to adult ecdysis (I1 to M4) for 103 larvae at 20 °C. **C** Duration of each stage of development for the larvae in **B**. **D** Coefficient of variation for the complete development (I1 to M4) and for each stage independently. Each dot represents the CV of an independent experiment. Statistics show only significant results of one-way ANOVA. **E** Pairwise comparison of the duration of the stages for individual larvae. The line represents the linear regression of the data forced through the origin (0,0). For each correlation, the Pearson *r* values are shown in the relevant graph. **F** Pairwise correlation matrix for all combinations of developmental stages, including *p* values when significant. In all cases * *p* < 0.05, ** *p* < 0.01, *** *p* < 0.001 and **** *p* < 0.0001

We quantified *C. elegans* postembryonic developmental progression to evaluate interindividual variability. Using a luminometry-based method, we determined the length of larval stages [11]. This method relies on the use of a *C. elegans* strain that constitutively expresses the *Photinus pyralis* luciferase. This enzyme catalyses the oxidation of luciferin, in a reaction that emits light. When luciferin is provided with the food during larval development, the larvae emit light only during intermolts, when they feed. With the formation of a buccal plug at the beginning of each molt, luciferin intake is prevented, and the bioluminescence reaction does not take place. Unlike other methods, this one allows measuring the duration of the molting period. As published elsewhere [6], we use a nomenclature that divides each larval stage into molt and intermolt (Fig. 1A). We thus used the measurements to determine the duration of each of the four complete larval stages (L1, L2, L3 and L4), as well as that of the intermolt and molt (i.e., L1 = I1+M1).

The average duration of development from hatching to adulthood for 103 larvae measured in six independent experiments at 20 °C was 50.67±1.95 h (Fig. 1B). This is similar to previous observations at the same temperature [12, 22, 23]. The average (±SD) duration of each stage of postembryonic development is 14.95±1.38 for I1, 2.38±0.18 for M1, 9.01±0.99 for I2, 2.17±0.17 for M2, 7.37±0.49 for I3, 2.19±0.14 for M3, 9.85±0.51 for I4 and 2.83±0.16 for M4 (Fig. 1C and Additional file 1: Fig. S1A). The coefficient of variation (CV) in the duration of the complete postembryonic development (from hatching to the end of M4) was 3.8% (Fig. 1D), lower than the 4.6% value recently measured by direct observation of movement of the N2 strain [13].

We also calculated the fraction of development in each larval stage by dividing the duration of each stage by the total duration of development. For the four larval stages (L1 to L4), we obtained the average values of 0.34, 0.22, 0.19 and 0.25 respectively (Additional file 1: Fig. S1B-C). These values are similar to those observed in multiple studies at different temperatures [3, 12, 13, 22, 24]. This means that the transgenic animals used for the luminometry assay conform to reported temporal structures of developmental timing of the wild-type strain, considered for both duration and variability.

Stage-by-stage analysis revealed that temporal precision differs between stages (Fig. 1D). The coefficient of variation is higher at the beginning of larval development, especially at L2, and lower at later stages, especially at L4. Furthermore, the coefficient of variation of the fractional duration showed a modest reduction compared to that of the total duration of each stage (Additional file 1: Fig. S1B-C, right panels), as has been observed elsewhere [13].

To further evaluate whether different developmental periods scale proportionally, we plotted the duration of the larval stages of individual larvae against each other, for all six possible combinations. Most combinations yield results that suggested a lack of correlation between the duration of larval stages (Fig. 1E). When we tested the correlation between each stage of larval development, we observed a non-significant negative correlation between I1 and each of the following intermolts and positive correlations when comparing late larval stages, especially between the third and fourth intermolts and molts (Fig. 1F). These observations suggest that correlation between the duration of the stages is limited to the last stages of development.

We tried to understand why we found a positive correlation only between the duration of late larval stages. These stages also show less variability than L1 and L2. Increased variability of L1 and L2 may be breaking the correlation between the duration of the stages. Since the larger variability of L1 and L2 has been recently observed using the N2 strain [13], we are inclined to think that this is a feature of *C. elegans* postembryonic development. Interindividual variability of developmental rate has been linked to maternal age [25]. Indeed, when we analysed stage-by-stage development of larva from mothers of different age, we observed that maternal age mainly impacts the duration of I1 and I2 (Additional file 1: Fig. 1D). Furthermore, maternal exposure to pheromones specifically delays I1 and molts of the progeny [26]. Similar to these two examples, other factors that generate interindividual variability might impact development in a stage-specific manner.

Our results contrast with other studies which found temporal scaling between the different stages of development [13, 27]. Our conditions of growth are different from other reports in that we grow the larvae in 96 well plates in liquid media, which could be a source of variability between the datasets. We would also like to reflect on the possibility that the correlation between the duration of larval stages would emerge from the combination of measurements of development from larvae raised at slightly different temperatures. We found that small variations in incubation temperature, as low as 0.85 °C, increase variability in postembryonic development comparable to that found in other studies (Additional file 1: Fig. S1E). Since the effect of temperature is similar in the different stages (especially when lacking resolution between molts and intermolts; see below), these small variations in temperature increase the correlation between the duration of stages. As an example, correlation increased when we combined the results from the original 103 larvae measured at 20 °C (T20) with a group of 17 larvae (T20.85) that reduced the average duration of development by ~30 min. These measurements (T20.85) come from an experiment set up in another luminometer than that of T20 (different piece of equipment of the same brand and model) which leads to a slightly elevated temperature at the plate that contains the larvae. The average duration of the T20.85 group could be obtained by an increase in experimental temperature of only 0.85 °C (as calculated by the temperature dependence of developmental rate, see below). When we added 18 more larvae (T21.25), the average duration of development changed by ~1 h. The average duration of the T21.25 group corresponds to a shift in temperature of 1.25 °C. In this case, we observed an additional increase in correlation (Additional file 1: Fig. S1E). The coefficient of variation of T20 plus T20.85 group is 4.71 %, similar to that found in previous works that report temporal scaling of development. The data of the three temperatures combined looks superficially like that of only the T20 group (Additional file 1: Fig. S1F). However, individual plotting of the three groups shows the acceleration of larval development with temperature (Additional file 1: Fig. S1G-H). This analysis suggests that estimates of interindividual variability in the duration of development might be easily influenced and obscured by variability due to small changes in temperature such as those that can occur in many labs. When reducing temperature-dependent variability, the duration of the stages of postembryonic development varies independently from each other. This means that progressing faster through one stage does not necessarily mean progressing faster for the following ones.

### Temperature dependence of larval development

**Fig. 2:**
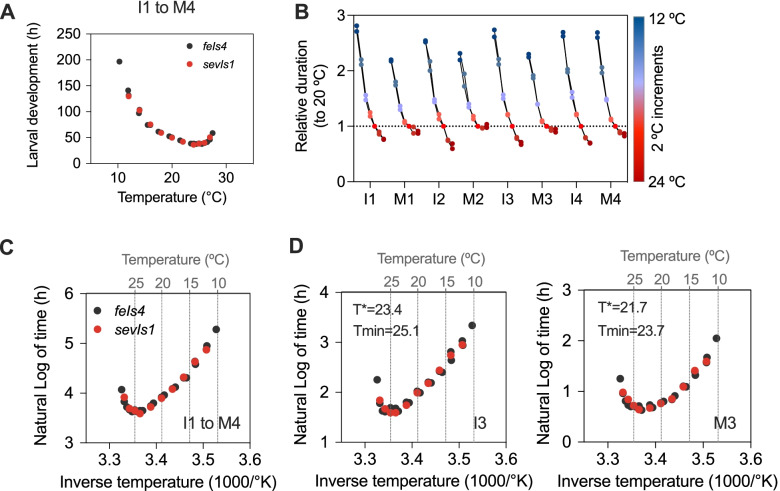
Temperature dependence of *C. elegans* postembryonic development. All panels except B represent the data for three datasets (two reporter strains/ two independent replicates; see methods section for more details). **A** Duration of larval development (I1–L4) at temperatures between 10 and 27 °C. **B** Duration of each stage relative to the duration at 20 °C for the two datasets with the same incubations temperatures (Datasets 2 and 3). **C** Arrhenius plot for the duration of development (I1 to M4). **D** Same representation as in **C** for two stages of development (I3 and M3)

A simple method to probe developmental timing in the nematode is comparison at various temperatures. The rate of development of poikilotherms correlates with external temperature. *Caenorhabditis elegans* larval development is accordingly temperature dependent [12, 27]. Recent studies have analysed developmental timing at three temperatures within a limited range (15–23 °C). We therefore analysed larval development under temperatures between 10 and 27 °C. The duration of the larval development spans from ~200 h at 10 °C to ~36 h at 24 °C. Above 24 °C, the duration of development lengthens (Fig. 2A). To compare the effect of temperature on each stage of development, we calculated the ratios of their duration at each temperature relative to that at 20 °C, the standard culture condition. We observed that the relative speed at extreme temperatures changed less for molts than for intermolts. The only exception is the fourth molt, which responded approximately to the same extent as intermolts (Fig. 2B).

Unlike postembryonic development, the duration of *C. elegans* embryonic development has been tested at temperatures between 4.5 and 30 °C. In that case, temperature dependence of different embryonic intervals follows the Arrhenius equation, which describes the relationship between temperature and the rate of first-order chemical reactions [28]. Although all the intervals tested followed Arrhenius within a range of temperatures, the durations of the different intervals do not scale proportionally with temperature [28], as also described for frog and fly embryos [29]. In order to perform the corresponding analysis for postembryonic development, we plotted the natural logarithm (ln) of the duration against the inverse of the temperature in Kelvin. In this representation, the data for an interval of temperature (~12 to 24 °C) becomes linear, which indicates fitting to the Arrhenius equation (Fig. 2C, Additional file 2: Fig. S2A). However, when we analysed each stage independently, differences between intermolts and molts were revealed. Namely, for the duration of the molts, the interval of temperatures that follows the Arrhenius equation was markedly reduced (Fig. 2D, Additional file 2: Fig. S2B, and Additional file 4: Table S1). We calculated two critical values, T* and Tmin, for larval development (Ls) and for the individual stages (Is and Ms; Additional file 4: Table S1). T* is the temperature where development deviates from the Arrhenius equation, and Tmin is the temperature that produces the fastest development [28]. For larval development, T* was 22.8 °C and Tmin was 24.5 °C, lower than the values for various stages of embryonic development. The calculation of T* showed that molts deviate from Arrhenius at lower temperatures than the corresponding intermolt (~ 1 °C). Accordingly, the temperature that sustained fastest development was lower in molts than in intermolts (Fig. 2D and Additional file 2: Fig. 2B), consistently with the analysis of relative durations. Since deviations from Arrhenius at high temperatures seem to be caused by the presence of nonideal behaviour of individual enzymes [29], this result suggests that the process of molting involves reactions that have a narrower range of optimal temperature.

**Fig. 3:**
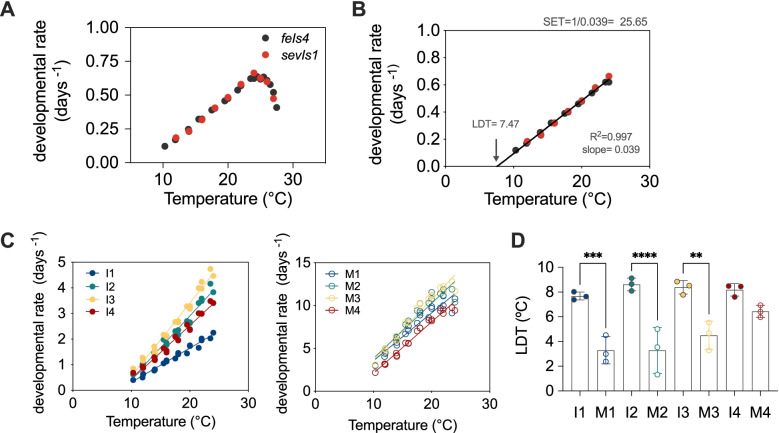
Postembryonic development deviates from developmental rate isomorphy. All panels except **B** represent the data for three datasets (two reporter strains/ two independent replicates; see methods section for more details). **A** Temperature dependence of developmental rate (1/days to complete larval development). **B** Calculation of LDT and SET values from the data within the linear range of temperature dependence (10 to 24 °C). **C** Temperature dependence of the developmental rate for each stage of development. **D** Lower developmental threshold (LDT) for each stage of development. Statistics show significant differences between the intermolt and molt of each stage except for I4–M4

Next, we analysed the variation of developmental rate, defined as the inverse of the duration of development in days, in response to changes in temperature. The increase of developmental rate with temperature is linear between 10 and 24 °C (Fig. 3A). This analysis, performed for temperatures below 24 °C, allows calculation of two values that define temperature dependence of development, namely the lower developmental threshold (LDT) and the sum of effective temperatures (SET) [30] (Fig. 3B). The LDT is defined as the *x*-intercept of the linear regression. Assuming linearity, the LDT would correspond to the lower bound of temperature at which development proceeds. For development as a whole, the LDT is ~7.5 °C. SET is defined as the inverse of the slope of the dependence between temperature and the rate of development (=1/[duration of development]) and is 25.65 day-degrees (Fig. 3B).

We then performed the same analysis for each stage of development (Fig. 3C) and calculated SET and LDT values. The SET values roughly reflect the duration of the larval stage (Additional file 2: Fig. S2C). LDT calculations yielded a less intuitive result, showing differences between intermolts and molts, although these were much less pronounced for the last larval stage (Fig. 3D). Furthermore, different LDTs among stages suggest departure from developmental rate isomorphy (DRI), which specifies that the proportion of total developmental time spent in a particular stage does not change with temperature [31, 32]. Since analysis of DRI requires the use of proportional data, we calculated the duration of each stage relative to the total duration of development (Additional file 2: Fig. S2D). When we plotted the relative durations against the temperature, we observed that they were not constant, suggesting non-isomorphic responses between stages of development. Molts and intermolts showed a different trend (Additional file 2: Fig. S2E). The slope of the relative durations indicates that the fraction of development spent in molting increased at higher temperatures, while the fraction of development occupied by intermolts decreased at high temperatures. The slope of each intermolt is significantly different from any of the molts. Furthermore, the deviations from isomorphy are larger at early stages of development, except for I1 (Additional file 2: Fig. S2F). When comparing only among intermolts, or among molts, there are statistically significant differences between I1 and I2, and between M1/M2 with M4. Ubiquity of intraspecific DRI has been questioned recently [31, 33] and our finding of different LDT for molts and intermolts also argues against DRI in *C. elegans* postembryonic development.

Altogether, three different types of analysis suggest that developmental stages differ in their response to temperature. Furthermore, since we tested a wide range of temperatures with high resolution (1–2 °C), we have been able to unveil relevant characteristics of development, such as the stage-specific adherence to and deviation from the Arrhenius equation, the calculation of the temperature that sustains fastest growth, and prediction of the minimal temperature that allows larval development.

### Food quantity and quality have stage-specific effects

**Fig. 4:**
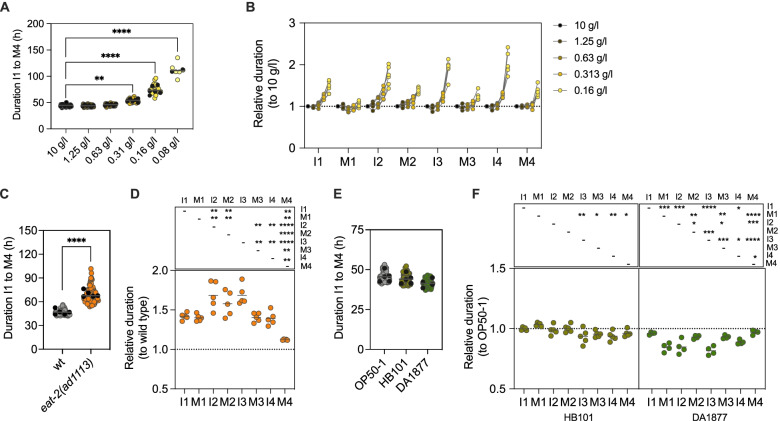
Food quantity and quality have stage-specific effects on postembryonic development. **A** Total duration of development at different concentrations of *E. coli* OP50-1, from 10 to 0.08 g/l. **B** Duration of each stage of development relative to that at the highest concentration of food. **C** Duration of development of the wild-type strain and the *eat-2(ad1113)* mutant. **D** Duration of each stage of development of the *eat-2* mutant relative to that of the wild-type. **E** Duration of development of the wild-type strain on HB101 and DA1877 diets. **F** Duration of each stage of development on HB101 and DA1877 diets relative to the duration on OP50-1. In **A**, **C** and **E**, grey or coloured markers show the values of individual animals and black dots represent the average of each experiment. The dots in **B**, **D** and **F**, represent relative durations calculated from the average of each experiment

We hypothesized that other perturbations would also affect different stages differently. Food quantity and quality affect the overall duration of *C. elegans* postembryonic development, but a detailed characterization of the effect on molts and intermolts has never been performed. We titrated the amount of the standard OP50-1 *Escherichia coli* diet provided to the larvae and measured developmental progression. Reduction of the OP50-1 concentration from 10 to 0.63 g/l did not have any effect on the duration of complete development or on the individual larval stages. However, below the concentration of 0.31 g/l of *E. coli* OP50-1, larval development started to show a significantly increased duration. At 0.08 g/l, a reduced fraction of animals (5/35) reached adulthood (Fig. 4A,B). We checked whether the duration of all the stages was reduced proportionally by calculating the ratios between the duration at each concentration relative to that at the highest concentration (10 g/l). The reduction of food to 0.16 g/l had a greater impact in the duration of I2, I3 and I4, each showing a larger than 2-fold increase in duration compared to the concentrated food source. I1, M2, M3 and M4 experienced around a 1.5-fold increase in duration in the same condition and M1 showed little variation between concentrations (Fig. 4B).

We also tested development of animals carrying the *eat-2(ad1113) *allele. *eat-2 *mutants have reduced pumping rates compared to the wild-type strain and are commonly used as a model for dietary restriction [34, 35]. The duration of complete development for the *eat-2(ad1113) *mutant was significantly increased compared to that of the wild-type (Fig. 4C). A detailed analysis of the duration of each stage in the mutant relative to wild-type showed that I2, M2 and I3 are the stages most affected by the reduction of pumping, while the duration of M4 is very similar to that of the wild-type (Fig. 4D).

Food quality also alters the duration of *C. elegans* development. We used two bacterial strains that are considered good food sources for *C. elegans* [19], *E. coli* HB101 and *Comamonas* DA1877. The effect of the *Comamonas* diet in the speed of development is mediated by vitamin B12, the levels of which are much higher in this bacterium than in *E. coli* OP50 [36]. While HB101 had only a mild, non-significant effect in the duration of complete development, the DA1877 diet reduced the duration of development by about 3 h (Fig. 4E). While the duration of the complete development is not significantly different, stage by stage analysis revealed that the duration of M1, I2 and I3 is significantly reduced by the *Comamonas *diet. Indeed, when we calculated the duration of development relative to the OP50-1 diet, M1, I2 and I3 showed a lower relative duration compared to the other stages (Fig. 4F).

Molts vary less than intermolts, both in response to temperature changes and to differences in food quantity, with the exception of M4 that shows the largest temperature sensitivity among the molts (Fig. 2B and Fig. 4B). These results show that food-related interventions have different effects than those induced by changes in temperature. Different food regimens are also distinct relative to each other. For instance, M1 is affected similarly to I1 by the *eat-2(ad113)* mutation. However, M1 is accelerated by the *Comamonas* diet to a larger extent than I1 (Fig. 4 D,F). This demonstrates that not all modulations of developmental rate by different food-related interventions are equal. Here, vitamin B12 from the *Comamonas* diet seems to impact preferentially processes in M1, I2 and I3. In summary, each intervention shows a stage-dependent signature in their modulation of developmental rate.

### Reduced insulin signalling has differential impact on larval stages

The mutant *daf-2(e1370)* is commonly used as a model for reduced insulin/IGF-1 signalling (IIS). The insulin signalling pathway promotes growth in the presence of nutrients. IIS is highly conserved and is a prominent, determinant regulator of ageing and lifespan in worms, flies and mammals, including humans [37]. DAF-2 is the *C. elegans* insulin receptor, whose activation triggers a phosphorylation cascade that eventually inhibits the entrance of the transcription factor DAF-16 to the nucleus. Low signalling through the IIS pathways reduces DAF-16 phosphorylation, allowing its translocation to the nucleus, where it activates transcription of target genes. The DAF-2 receptor is activated by the binding of agonist insulin-like peptides (ILPs) produced in response to the presence of food (reviewed in [38]).

The allele *daf-2(e1370)* shows developmental delays, especially at the L2 stage [11, 21]. Furthermore, *daf-2(e1370)* is widely described as a thermosensitive allele. *daf-2(e1370)* forms dauer larvae at 25 °C but not at 15–20 °C. However, other phenotypes, such as lifespan extension, are present at 15 °C, suggesting that *e1370* is not thermosensitive in the classical sense but that the dauer phenotype is temperature dependent (reviewed in [39]).

**Fig. 5:**
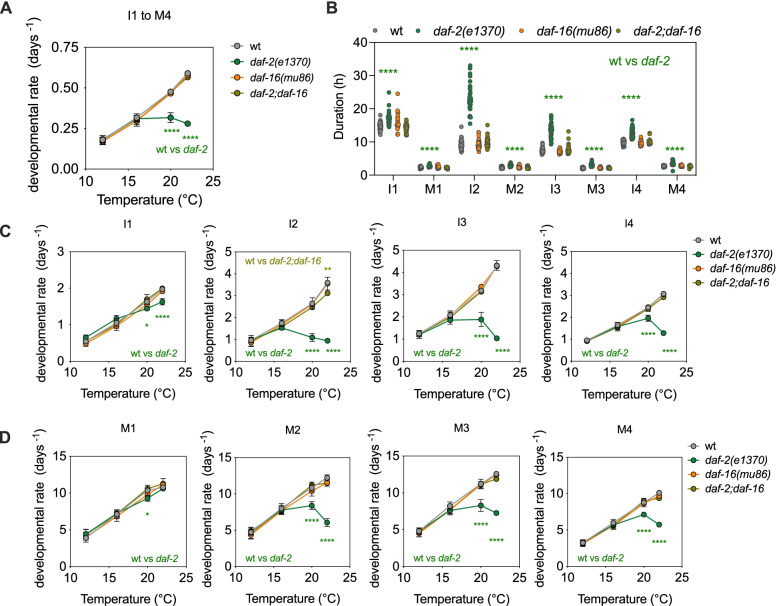
Reduced insulin signalling predominately impacts I2 through M3. **A** Developmental rate of the wild-type and the mutants *daf-2(e1370)*, *daf-16(mu86)* and *daf-2(e1370);daf-16(mu86)* at 12, 16, 20 and 22 °C. **B** Duration of each stage of development at 20 °C for the four strains. Each dot represents the value for a single animal. Developmental rate of intermolts (**C**) and molts (**D**) at 12, 16, 20 and 22 °C, for the wild-type and mutant strains. **A–D** Statistics show two-way ANOVA. All strains were compared to the wild-type, but only significant differences are shown

We thus decided to investigate temperature sensitivity of the developmental delay phenotype of *daf-2(e1370)*. This experiment provides an outstanding opportunity to assess the combined effect of both temperature and food sensing. We tested *daf-2(e1370), daf-16(mu86)* and the double mutant *daf-2(e1370);daf-16(mu86)* at 12, 16, 20 and 22 °C. The developmental rate of *daf-2(e1370)* increases between 12 and 16 °C, being indistinguishable from that of the wild-type. We observed no increase, however, in developmental rate between 16 and 20 °C, nor at 22 °C (Fig. 5A). The *daf-16* and *daf-2;daf-16* mutants developed as wild-type animals at all temperatures, indicating that DAF-16 is necessary for this *daf-2* phenotype (Fig. 5A). This might suggest that *daf-2* mutants are insensitive to temperature changes above 16 °C, but we know that the developmental delay of *daf-2* is stage dependent at 20 °C [11, 21] (Fig. 5B). We therefore analysed the effect of temperature on developmental rate of each stage independently. The developmental rate of the early stages, I1 and M1, increases in the mutant strains as in the wild-type, while later larval stages increased to a lower extent in the *daf-2* mutant relative to the wild-type. The most extreme difference in phenotype is found in the I2 stage, where developmental rate of the *daf-2* mutant was reduced between 16 and 20 °C. Furthermore, for L2 and L3, molts are less affected by the mutation than intermolts (Fig. 5C). Again, these phenotypes are DAF-16 dependent, as the double *daf-2;daf-16* mutants show the same developmental rate as the wild-type. We also noticed temperature dependent differences in the progression of development of the mutants. At 12 °C, the successful completion of larval development required DAF-16, while at 22 °C, activation of DAF-16 in the *daf-2(e1370)* mutant prevented the progression of development at different stages (Additional file 3: Fig. S3).

The overall developmental delay in these mutants could be explained by thermosensitivity of the *e1370* mutation, as the phenotype is only observed at high temperatures. However, the stage-dependent effect of the *daf-2(e1370)* mutation at 20 °C points to a different contribution of the DAF-2 receptor (via DAF-16 transcription factor function) to the progression of each stage of development.

Our results suggest that lower IIS per se does not lead to developmental delays. Rather, in some larval stages, low IIS and temperatures above 20 °C leads to an activation of DAF-16 that extends the duration of the stage. Stage-specific extension could be the consequence of the activation of alternative developmental programs. The extended L2 observed in *daf-2(e1370) *confers resistance to the nicotinic acetylcholine receptor agonist DMPP (dimethylphenylpiperazinium) in a DAF-16-dependent manner. However, analysis of DMPP resistance of multiple *daf-2* alleles revealed that this phenotype does not correlate with their dauer phenotype [21]. This means that IIS can individually impact the different developmental stages, altering the processes occurring during them.

**Fig. 6:**
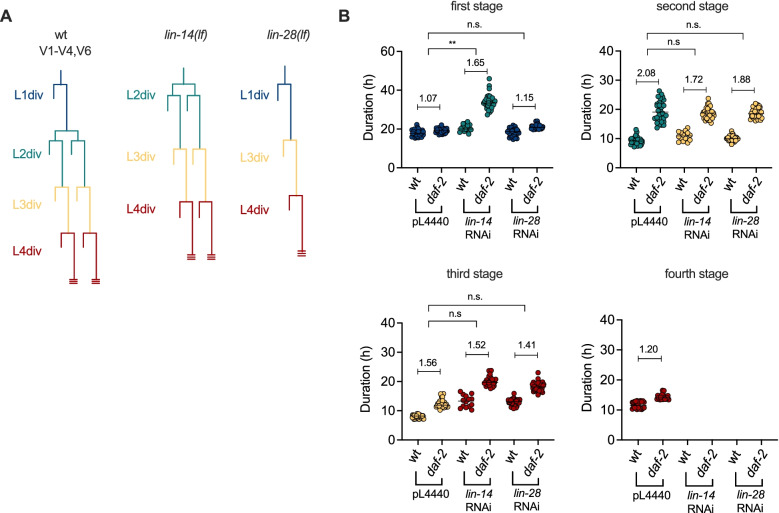
Stage-specific division patterns determine the duration of the larval stages. **A** Diagram of V1–V4,V6 seam cell divisions in the wild-type strain and *lin-14* and *lin-28* lack-of-function mutants. **B** Duration of each stage of larval development. The stages are plotted as first, second, third or fourth regardless of the cell fates expressed in the stage, which are indicated by the colour of the dots, as shown in **A**. The numbers inside the plots show the *daf-2* to wild-type ratio of the duration of each stage. The results from statistical testing refer to the differences between the ratios of each different RNAi compared to the PL4440 control. The duration of all *daf-2* stages is significantly different from that of the wild-type (not shown), as measured by *t*-test

The duration of the L1 stage is especially insensitive to low insulin signalling. This could be a consequence of this stage being the first after hatching. Since the effect of maternal provisioning to the embryos is strongest at L1 (Additional file 1: Fig. S1D), it could be that this provisioning in the newly hatched larvae is sufficient to maintain the wild-type L1 duration. Alternatively, it is also possible that the specific cellular events taking place during L1 are independent of insulin signalling. To investigate this, we decided to alter the pattern of postembryonic cell divisions and analyse the effect on the timing of the larval stages. Using RNAi, we knocked down the expression of the heterochronic genes *lin-14* and *lin-28*, which are respectively involved in the determination of L1 and L2 cell fates, during the development of the *daf-2(e1370)* mutant. In *lin-14* loss-of-function mutants, the blast cells that divide during postembryonic development skip the L1-specific cell fates so that hatching is followed by the L2-specific divisions. *lin-28* mutants show normal L1 divisions but premature activation of the L3 fates, skipping the L2 divisions (Fig. 6A) [15, 40].

In the previous experiments, the *daf-2(e1370)* mutation provoked an extension of L2, L3 and L4, but not L1. When we treated *daf-2(e1370)* larvae with *lin-14* RNAi, the first observable stage, corresponding to the L2 cell fates, shows a significant extension (Fig. 6B), proving that larval development can be sensitive to insulin signalling immediately after hatching. Treatment with *lin-28* RNAi, however, did not alter the duration of the first stage, which corresponds to the L1 divisions, as in the control (Fig. 6B). The differences between *daf-2* to wild-type ratios of the duration for the rest of the stages do not show significant differences between the control and the RNAi treatments. The *daf-2* mutation provoked an extension of the stages corresponding to L3 and L4 divisions in both *lin-14* and *lin-28* RNAi treatments, as occurs when these genes are knocked down in the wild-type background. Therefore, our results suggest the possibility that the L1 divisions specifically are insensitive to low insulin signalling, since *lin-14* RNAi treatment, which suppresses L1 divisions, leads to extension of the first stage after hatching (Fig. 6B). This result indicates that the duration of different larval stages may be defined by the cellular events taking place during that stage.

## Discussion

Despite the marked repetitive nature of the processes, each developmental stage features specific events. While L1 is dominated by division of neuronal lineages, L2 has a key role in determination of progression to dauer or L3 larva, the L3 stage is marked by development of the somatic gonad, and L4 by initiation of meiosis of germ cells that differentiate into mature sperm. Furthermore, nutritional checkpoints are present at the beginning of each larval stage [1] (reviewed in [41, 42]). These are only some of the stage-specific events during larval development but, with this in mind, it seems remarkable that the duration of the stages is so similar. A recent work has revealed coupling between growth rate and developmental timing [43]. The authors suggest two possible non-exclusive mechanisms to achieve this coupling. First, growth rate could impact a developmental clock. As a second mechanism, molting could be triggered by mechanical forces provoked by stretching of the cuticle, as also suggested recently [44]. This mechanism provides a plausible explanation for the similar duration of the stages that is independent of a developmental clock. However, some recent observations seem to challenge this connection between body size and developmental timing, as growth-arrested *lin-42* mutants continued development after they ceased increasing size [27].

Our results suggest that stage-specific events confer a different duration to each stage. Ranging from interindividual variability to the response to temperature and diet, our measurements of development reveal deviations from proportionality in the duration of the larval stages. Although there is a general trend that molts are less responsive than intermolts, the stages impacted by the various interventions are different. Regarding temperature, besides superficial proportionality of developmental stages, changes in temperature affect preferentially intermolts (Fig. 2B). With increasing temperatures, the fraction of time devoted to intermolts decreases while that dedicated to molts increases (Additional file 2: Fig. S2D-F). To unveil this complexity, we have tested three types of analysis that all point to different response to temperature of the developmental stages. The three methods of analysis show that larval stages do not scale proportionally with temperature. This finding is not surprising, as development is a complex process involving multiple reactions. A recent work found that, for frog and fly embryonic development, deviations from Arrhenius at high and low temperatures are explained to some extent by the coupling of multiple reactions, and to a larger extent by individual non-idealized rate-limiting processes [29]. Nevertheless, the response to temperature has the highest degree of proportionality among those we have tested. We speculate that this could contribute to increase interindividual proportionality among larvae, as addition of a small set of larvae raised at slightly different temperature considerably increased proportionality of the sample.

Regarding food quantity and quality, the differences between stages are varied and intervention dependent. This is in accordance with the effect of dietary restriction (DR) on other life traits as lifespan, where different DR methods act through different pathways yielding different effects on longevity [45]. While food titration and the use of pumping-defective mutants are considered somehow similar interventions, their effect in developmental timing can be completely different. For instance, while M2 shows similar response to food titration compared to other stages, its relative duration in the *eat-2* mutant is largely increased, comparable to that of I2 and I3 (Fig. 4B,D). While the *eat-2* mutation impacts all stages except for M4, the DA1877 diet affects mainly M1, I2 and I3 (Fig. 4D,F). Our data suggest that each intervention preferentially impacts events at different stages thus changing the duration of the stage. It will be interesting to analyse if this stage-dependent modulation depends on the nutritional checkpoint already described at the end of the molts [1] or whether the slowdown of the stages is due to other nutrient-sensitive events. Another feature that seems to be conserved across interventions is that I1 is less sensitive to changes. This could be a trivial consequence of being the first stage in our assay, and therefore newly exposed to the experimental conditions. It might also be possible that I1 is fundamentally different in that part of the resources to sustain this stage might come from embryonic provisioning. However, we have observed that the insensitivity of L1 to low insulin signalling can be bypassed when animals are treated with *lin-14* RNAi. This suggests the possibility that the duration of this stage is linked to the L1-specific cell fates.

## Conclusions

Altogether, our results provide strong evidence that the duration of each stage of postembryonic development is differentially affected by changes in environmental conditions, showing that their duration is differentially regulated. Our results suggest that multiple signals contribute to set the duration of development by independently impacting the different stages of the process.

## Methods

### C. elegans maintenance and strains

We cultured stock animals according to standard methods [46], maintaining them at 20 °C on nematode growth medium (NGM) with a lawn of *Escherichia coli* OP50-1. The only exception was one of the datasets performed to analyse the effect of temperature (PE254 *feIs4*, Dataset 1), for which the animals were maintained to 18 °C prior to the analysis of development at different temperatures. A detailed description of the strains used in this study is included in Additional file 4: Table S2.

### Bacterial strains

For the experiments where we altered the food source or performed RNAi treatment by feeding, we used the bacterial strains detailed Additional file 4: Table S2.

### Luminometry of single worms

We measured developmental timing using a bioluminescence-based method [11]. For the experiments initiated with embryos, we first obtained age-matched embryos by transferring 10–15 gravid hermaphrodites to a fresh NGM plate and allowing them to lay eggs over a period of approximately 2 h. Then, using an eyelash we transferred individual embryos to the wells of a white 96-well plate containing 100 μl of S-basal (including 5 μg/ml cholesterol) with 200 μM Luciferin. For the experiment initiated from arrested L1 (Figs. 2 and 3, strain PE254 *feIs4*, Dataset 1), we treated gravid adults with alkaline hypochlorite solution to obtain embryos and adjusted the concentration 20 embryos/μl of M9 buffer. The embryos were incubated overnight at 20 °C, with gentle shaking, leading to hatching and arrest at the L1 stage. Then, synchronized L1s where diluted in M9 buffer to allow pipetting of individual larvae to the wells of a white 96-well plate containing 100 μl of S-basal (including 5 μg/ml cholesterol) with 200 μM Luciferin, as before. After all embryos or larvae were placed in the wells, we added 100 μl of S-basal containing 20 g/l *E. coli* OP50-1, except when stated otherwise. We alternated the samples across the plate to avoid local effects (i.e., temperature of the luminometry reader). After preparation of the plate, we sealed the plates using a gas-permeable membrane. The plate was introduced in the luminometry reader (Berthold Centro XS3), which is placed inside a cooled incubator (Panasonic MIR-254) to allow temperature control. We measured luminesce for 1 s at 5-min intervals, until animals reached adulthood.

### Synchronization of mothers

To test the effect of maternal age in larval development, we obtained embryos from mothers in their first, second and third day of egg laying. We prepared synchronized populations allowing 20 gravid adults to lay eggs on NGM plates for 2 h. These synchronized populations were prepared approximately 136, 112 and 88 h before the experiment to obtain mothers in their third, second and first day of egg laying, respectively. The embryos from these mothers were used to initiate the luminometry experiments as detailed above, to measure postembryonic development.

### Temperature regulation

We measured the temperature in the plate using a data logger Thermochron iButton DS1921G (Maxim Integrated). The temperature in the plate is ~3.5 °C higher than that set at the incubator, due to the production of heat from the luminometer. All temperatures shown in the data correspond to the temperature experienced by the larvae.

For the evaluation of the effect of temperature on larval development, the animals were shifted from the maintenance temperature to the experimental temperature at the beginning of postembryonic development.

### Preparation of bacterial cultures

All strains were first grown overnight at 37 °C, shaking in LB medium with 100 μg/ml Streptomycin. Then, we diluted 1:10 in fresh LB and incubated for an additional 2.5 h. We transferred 100 ml of the culture to two 50-ml tubes and centrifuged 10 min at 4000*g* at 4 °C. We washed the bacterial pellet with 25 ml of S-basal and centrifuged again in the same conditions. After removal of the supernatant, we weighted the wet pellet and adjusted to 20 g/l using S-basal. For the experiments with different amounts of food, we performed serial dilutions to reach the desired concentrations.

### RNAi treatments

To knockdown *lin-14* and *lin-28*, we performed RNAi treatments with the corresponding clones from the Vidal library. We measured development on the second generation treated with RNAi to increase the efficiency of the treatment. To grow the first generation on NGM plates, we added 200 μl of an overnight culture of RNAi or control bacteria on NGM plates with 1 mM IPTG and 100 μg/ml Ampicillin. We let the bacterial lawn dry and incubated the plates for 5 h at 37 °C and overnight at room temperature. We transferred 10 gravid adults of the strain MRS387 or MRS434 per plate and let them lay eggs for ~1 h before removing them from the plates. We grew them for 4 days (MRS387, wild-type background) or 5 days (MRS434, *daf-2* mutant background) at 20 °C. The embryos produced by these treated animals were transferred to independent wells of a 96-well plate containing 100 μl of S-basal, to measure development as described above. The only difference was that, after all embryos or larvae were placed in the wells, we added 100 μl of S-basal containing 20 g/l of the control bacteria or the corresponding RNAi clones, 2 mM IPTG and 200 μg/ml Ampicillin. The final concentration of these components was 10 g/l of bacteria, 1 mM IPTG and 100 μg/ml Ampicillin. To prepare the 20 g/l bacterial suspension, we proceeded as explained above, but after incubation of the diluted culture for 3 h at 37 °C, we added IPTG to 1 mM and incubated for an additional 2 h. Efficiency of the *lin-14* and *lin-28* treatments was confirmed by the presence of only three larval stages in the luminometry prolife. For these two conditions, larvae with four molts were excluded from the analysis.

### Summary of experimental replicates and number of animals

Figure 1 and Additional file 1: Fig. S1 A, B and C contain measurements from 103 larva in six independent experiments. Additional file 1: Fig. S D represents data from three independent experiments. The number of animals for each condition is 58 (day 1), 62 (day 2) and 48 (day 3). In Additional file 1: Fig. S1 E-H, to the 103 larvae analyses in Fig. 1, we added data from 17 and 18 larvae from two additional experiments.

Figures 2 and 3 include the datasets detailed below. Each dataset contains one experiment at each of the indicated temperatures.

Dataset 1: strain PE254 feIs4_Replicate 1. The number of larvae per condition (temperature) is 31 (10.3 °C), 26 (11.9 °C), 24 (13.9 °C), 19 (15.5 °C), 27 (17.5 °C), 33 (19.5 °C), 33 (21.5 °C), 26 (23.5 °C), 37 (24.5 °C), 18 (25.5 °C), 22 (26.5 °C) and 37 (27.5 °C).

Dataset 2: strain PE254 feIs4_Replicate 2. The number of larvae per condition (temperature) is 17 (12 °C), 14 (14 °C), 21 (16 °C), 19 (18 °C), 18 (20 °C), 22 (22 °C), 15 (24 °C), 19 (25 °C), 22 (26 °C) and 21 (27 °C).

Dataset 3: strain MRS387 sevIs1_Replicate 2. The number of larvae per condition (temperature) is 19 (12 °C), 18 (14 °C), 17 (16 °C), 20 (18 °C), 18 (20 °C), 16 (22 °C), 21 (24 °C), 12 (25 °C), 21 (26 °C) and 18 (27 °C).

Figure 4A shows measurement from the following number of larvae 35, 40, 40, 33, 26 and 6 for the different amounts of food, from 10 to 0.08 g/l of OP50-1. Figure 4B shows data from the same larva, this time as relative duration. In Fig. 4B, we did not include the conditions with 0.08 g/l OP50-1 due to the low number of larvae that reached adulthood. These larvae were distributed in six independent replicates. Figure 4 C and D contain results from 67 wild-type and 84 *eat-2(ad1113)* larvae, in five independent experiments. Figure 4 E and F represent the results from five experiments for HB101 and 4 experiments for DA1877, with a total of 61 larvae for OP50-1, 61 for HB101 and 59 DA1877.

Figure 5 A–D include data from three independent experiments at each temperature. The number of larva for each condition is as follows: at 12 °C, 41 (wt), 46 (*daf-16*), 47 (*daf-2*) and 49 (*daf-2;daf-16*); at 16 °C, 61 (wt), 39 (*daf-16*), 61 (*daf-2*) and 49 (*daf-2;daf-16*); at 20 °C, 40 (wt), 34 (*daf-16*), 40 (*daf-2*) and 52 (*daf-2;daf-16*); and at 22 °C, 53 (wt), 57 (*daf-16*), 56 (*daf-2*) and 59 (*daf-2;daf-16*).

Figure 6B contains measurements from 33 (wt; pL4440), 14 (wt; *lin-14* RNAi), 32 (wt; *lin-28* RNAi), 30 (*daf-2*; pL4440), 30 (*daf-2*; *lin-14* RNAi) and 31 (*daf-2*; *lin-28* RNAi) larvae, in three independent experiments.

### Data analysis and statistics

We analysed luminometry data as previously described [11]. Shortly, we determined the timing of the molts to calculate the duration of each stage. We first calculated the moving average of the data in a time window of 12 h. Then, we converted the raw values of luminescence to binary using as a threshold the 75% of a 12-h moving average. To evaluate the data for onset and offset of molting, we detected the transitions in the binarized data. Transitions from 1 to 0 correspond to onset of the molt and transitions from 0 to 1 correspond to offset of the molt.

To test for differences in the duration of the complete development compared to the wild-type in Fig. 4 A and E, we used the one-way ANOVA followed by Dunnett’s multiple comparisons test. For the comparison in Fig. 4C, we used an unpaired *t*-test. To compare among stages in Figs. 1 and 2D and S2F, we performed one-way ANOVA followed by Tukey’s test. In all cases, * means *p* > 0.05, ** *p* > 0.01, *** *p* > 0.001 and **** *p* > 0.0001. Graphs and statistics were performed on Prism 9.

### Arrhenius analysis

We used the bootstrapping regression method to obtain critical values, T* and Tmin, as described before [28]. This statistical approach allowed us to estimate the errors associated with the interval fit regression parameters for the complete development and the individual stages. At each stage, the exponential range of the data was fit by the Arrhenius equation:

$$\mathrm{Time}\left({\mathrm{event}}_2\ {event}_1\right)=A\ \exp \left({E}_a/\mathrm{RT}\right)$$

To include the high-temperature points, the equation defined by [28], which contains an additional term to include the high-temperature data, was used:

$$\mathrm{Time}\left({\mathrm{event}}_2-{event}_1\right)={A}_1\exp \left({E}_1=\mathrm{RT}\right)+{A}_2\exp \left({E}_2=\mathrm{RT}\right)$$

Once the terms necessary for the calculation of the Arrhenius fit were defined, the mean and SD of T* and Tmin were estimated from fits after bootstrapping with 1000 random resamples using a custom-made script in R software.

To calculate the Arrhenius interval, that is, the interval of temperatures that fit the Arrhenius equation, we proceeded as in [28]. Shortly, we calculated the slope of a linear fit to the three data points centred around 20 °C. Then, we added one by one the data point from adjacent temperatures to the fit and calculated the new slopes. If the new slope shows below 10% deviation from that of the starting interval, the added temperature is included in the Arrhenius interval.

## Supplementary Information

**Additional file 1: Figure S1.** (Related to Fig. 1). Interindividual variability in larval development. (A) Cumulative distribution of the beginning (ON) and end (OFF) of the four molts of postembryonic development for 103 larvae. (B) Duration and coefficient of variation (CV) for the duration of each larval stage. (C) Fraction of development dedicated to each larval stage and coefficient of variation of these values for 103 larvae. (D) Duration of each stage of development for larvae with different maternal age. Day 1, day and day 3 correspond to mothers on their first, second and third days of egg laying. (E) Correlation matrixes for each combination of larval stages for the 103 larvae in Fig. 1, and after adding 17 and 18 larvae from plates that displace the average duration by 30 min and 60 min respectively. (F) Duration of each intermols and molt the three groups combined (T20+ T20.85 + T21.25). (G) Duration of each larval stage of each of the three groups (T20 / T20.85 / T21.25). (H) Pairwise comparison of the duration of L1 and L3 of the three datasets.

**Additional file 2: Figure S2.** (Related to Figs. 2 and 3): Temperature dependence of developmental stages. (A) Linear regression showing fitting within the Arrhenius interval of the Dataset 3. (B) Arrhenius plots for each intermolt and molt, showing the values of T* and Tmin calculated for Dataset 3. (C) SET values for each larval stage, calculated for each of the three Datasets. (D) Fraction of development devoted to each stage of development at the temperatures within the linear range defined in Fig. 2F. (E) Average fraction of development devoted to each intermolt (left) and molt (right) for Dataset 3 (F) Representative plots showing the slope of the linear regression of the fractional durations for each stage of development. The three dots for each stage correspond to each of the three datasets. Statistics show significant differences among intermolts, and among molts. Additionally, each intermolt is significantly different from each of the molts.

**Additional file 3: Figure S3.** (Related to Fig. 5). Fraction of animals that completes each stage of development at the different temperatures for the wild-type and the *daf-2(e1370)*, *daf-16(mu86)* and *daf-2; daf-16 *mutants.

**Additional file 4: Table S1.** Values of T*, Tmin and Arrhenius interval for the data of the results of Dataset 3, performed with the reporter *sevIs1*. The calculation of these values is described in the methods. **Table S2.** Strains used in this study [47–50].

## Data Availability

The datasets supporting the conclusions of this study are available in the Mendeley data repository, [DOI: 10.17632/phxjk4624v.1] [51].
