# Supplementary figures and images for: Deviations from temporal scaling support a stage-specific regulation for C. elegans postembryonic development
Source: BMC Biol. 2022 Apr 27;20:94. doi: 10.1186/s12915-022-01295-2 (PMC9047341; doi:10.1186/s12915-022-01295-2)

**A**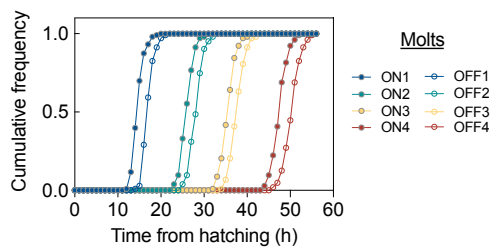**B**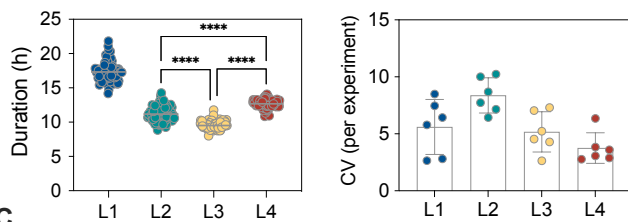**C**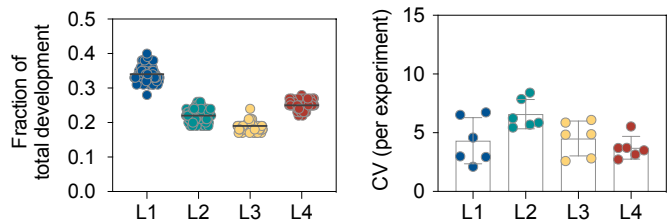**D**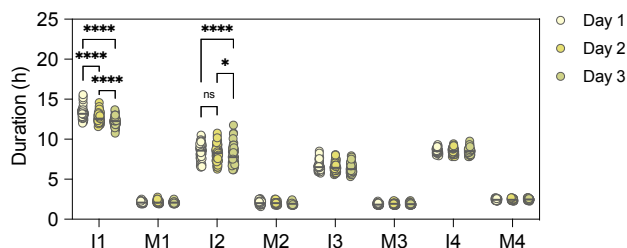**E**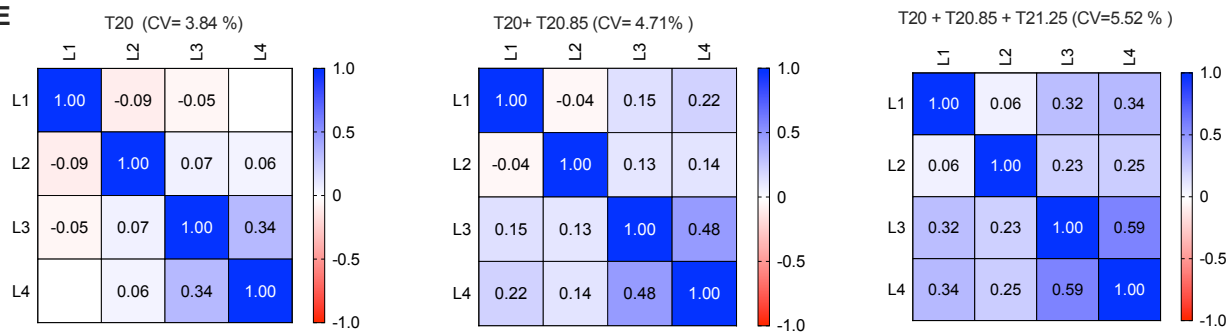**F**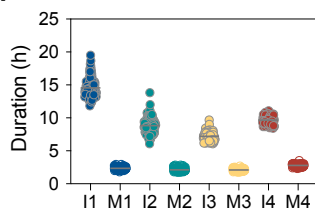**G**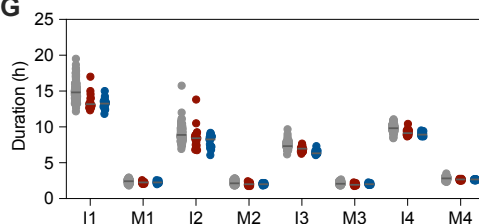**H**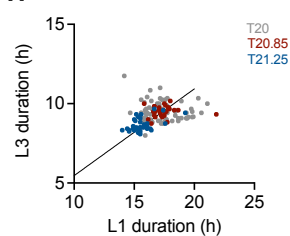

Supplement: Supplementary file 1 — Additional file 1: Figure S1. (Related to Fig. 1). Interindividual variability in larval development. (A) Cumulative distribution of the beginning (ON) and end (OFF) of the four molts of postembryonic development for 103 larvae. (B) Duration and coefficient of variation (CV) for the duration of each larval stage. (C) Fraction of development dedicated to each larval stage and coefficient of variation of these values for 103 larvae. (D) Duration of each stage of development for larvae with different maternal age. Day 1, day and day 3 correspond to mothers on their first, second and third days of egg laying. (E) Correlation matrixes for each combination of larval stages for the 103 larvae in Fig. 1, and after adding 17 and 18 larvae from plates that displace the average duration by 30 min and 60 min respectively. (F) Duration of each intermols and molt the three groups combined (T20+ T20.85 + T21.25). (G) Duration of each larval stage of each of the three groups (T20 / T20.85 / T21.25). (H) Pairwise comparison of the duration of L1 and L3 of the three datasets. [file 12915_2022_1295_MOESM1_ESM.pdf]

**A**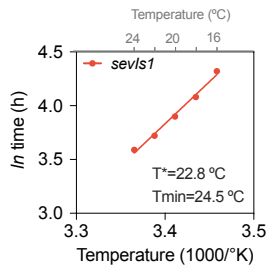**B**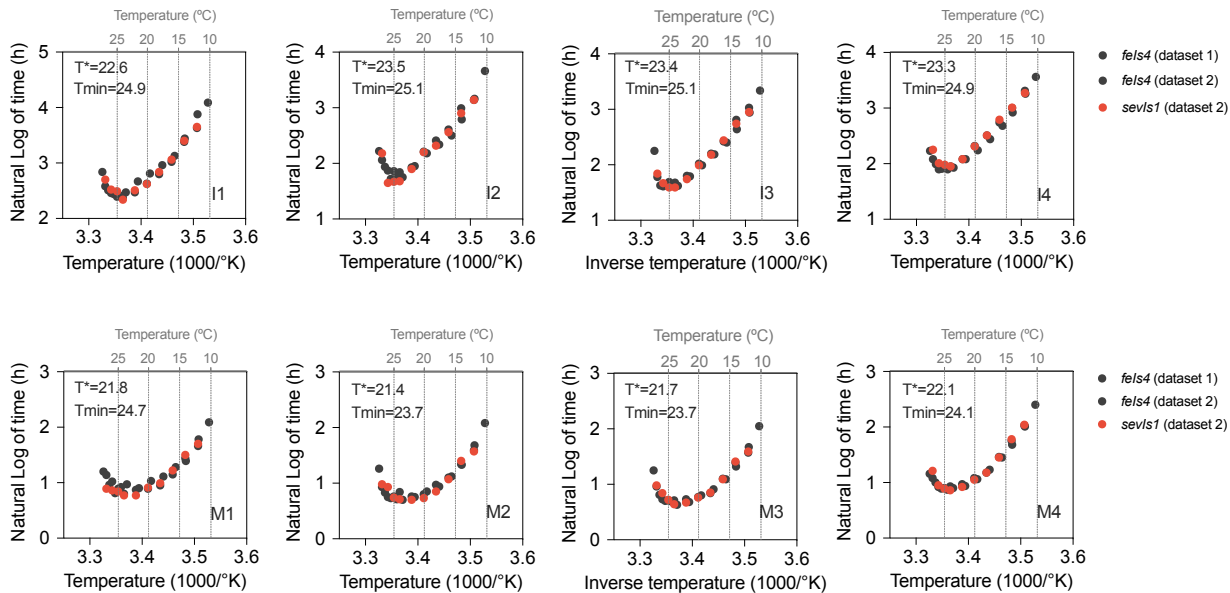**C**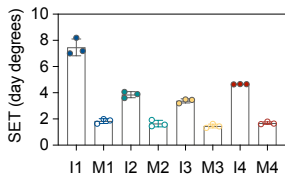**D**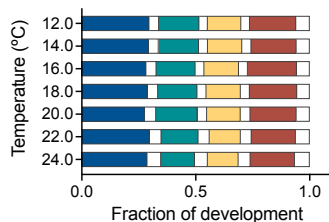**E**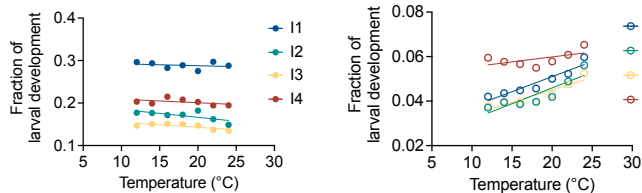**F**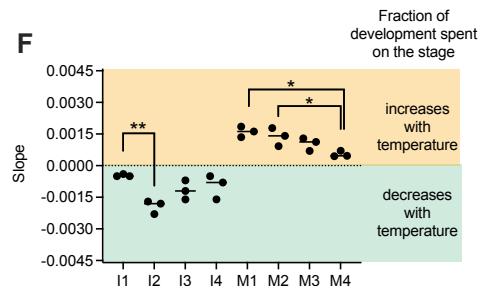

Supplement: Supplementary file 2 — Additional file 2: Figure S2. (Related to Figs. 2 and 3): Temperature dependence of developmental stages. (A) Linear regression showing fitting within the Arrhenius interval of the Dataset 3. (B) Arrhenius plots for each intermolt and molt, showing the values of T* and Tmin calculated for Dataset 3. (C) SET values for each larval stage, calculated for each of the three Datasets. (D) Fraction of development devoted to each stage of development at the temperatures within the linear range defined in Fig. 2F. (E) Average fraction of development devoted to each intermolt (left) and molt (right) for Dataset 3 (F) Representative plots showing the slope of the linear regression of the fractional durations for each stage of development. The three dots for each stage correspond to each of the three datasets. Statistics show significant differences among intermolts, and among molts. Additionally, each intermolt is significantly different from each of the molts. [file 12915_2022_1295_MOESM2_ESM.pdf]

12 °C

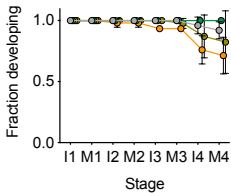

16 °C

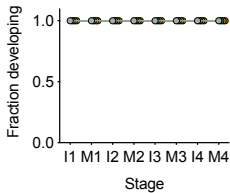

20 °C

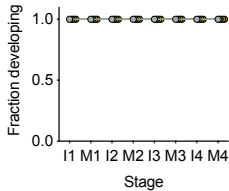

22 °C

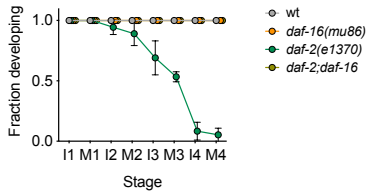

Supplement: Supplementary file 3 — Additional file 3: Figure S3. (Related to Fig. 5). Fraction of animals that completes each stage of development at the different temperatures for the wild-type and the daf-2(e1370), daf-16(mu86) and daf-2; daf-16 mutants. [file 12915_2022_1295_MOESM3_ESM.pdf]
