# Supplementary material for: Deviations from temporal scaling support a stage-specific regulation for C. elegans postembryonic development
Source: BMC Biol. 2022 Apr 27;20:94. doi: 10.1186/s12915-022-01295-2 (PMC9047341; doi:10.1186/s12915-022-01295-2)
Supplement: Supplementary file 4 — Additional file 4: Table S1. Values of T*, Tmin and Arrhenius interval for the data of the results of Dataset 3, performed with the reporter sevIs1. The calculation of these values is described in the methods. Table S2. Strains used in this study [47–50]. [file 12915_2022_1295_MOESM4_ESM.pdf]

Table S1.

	T* (ºC)	Tmin (ºC)	Arrhenius interval
**M1-M4**	22.83 ± 0.45	24.54 ± 0.19	16 ºC – 24 ºC
**I1 to M4**	22.78 ± 0.50	24.48 ± 0.22	16 ºC – 24 ºC
**I1**	22.63 ± 0.93	24.90 ± 0.61	16 ºC – 24 ºC
**M1**	21.76 ± 1.00	24.69 ± 0.64	18 ºC – 22 ºC
**I2**	23.48 ± 0.39	25.14 ± 0.35	16 ºC – 26 ºC
**M2**	21.36 ± 1.11	23.73 ± 0.37	18 ºC – 22 ºC
**I3**	23.37 ± 0.68	25.06 ± 0.18	16 ºC – 24 ºC
**M3**	21.73 ± 0.92	23.74 ± 0.33	18 ºC – 22 ºC
**I4**	23.32 ± 0.31	24.95 ± 0.14	16 ºC – 22 ºC
**M4**	22.15 ± 0.69	24.11 ± 0.28	18 ºC – 22 ºC

Table S2.

	**Source**
**Bacterial strains**	
E. coli: OP50-1	*Caenorhabditis* Genetics Center
E. coli: HB101	*Caenorhabditis* Genetics Center
Comamonas sp.: DA1877	*Caenorhabditis* Genetics Center
E. coli HT115(DE3) pL4440	Rual et al., 2004 [47]
E. coli HT115(DE3) *lin-14* RNAi	Rual et al., 2004 [47]
E. coli HT115(DE3) *lin-28* RNAi	Rual et al., 2004 [47]
***C. elegans* strains**	
PE254 *feIs4 [Psur-5::luc+::gfp; rol-6 (su1006)]V*	Lagido et al., 2008 [48]
MRS387 *sevIs1 [Psur-5::luc+::gfp]X*	Olmedo et al., 2020 [49]
MRS424 *daf-16(mu86)I; sevIs1 [Psur-5::luc+::gfp]X*	Olmedo et al., 2020 [49]
MRS434 *daf-2(e1370)III; sevIs1 [Psur-5::luc+::gfp]X*	Olmedo et al., 2020 [49]
MOL56 *daf-2(e1370)III; daf-16(mu86)I; sevIs1[Psur-5::luc+::gfp]X*	Olmedo et al., 2020 [49]
MRS307 *eat-2(ad1113) II; feIs4[sur-5::luc+::gfp; rol-6(su1006)]V*	Rodríguez-Palero et al., 2018 [50]
